# Supplementary material for: Mechanisms governing the pioneering and redistribution capabilities of the non-classical pioneer PU.1
Source: Nat Commun. 2020 Jan 21;11:402. doi: 10.1038/s41467-019-13960-2 (PMC6972792; doi:10.1038/s41467-019-13960-2)
Supplement: Supplementary file 7 — Source data [file 41467_2019_13960_MOESM7_ESM.zip › Source_Data/FigureS5/FigureS5A_MotifsCommonPeaks/homerResults/motif4.similar.html]

motif4

## Information for motif4

A
G
T
C
A
G
C
T
A
G
C
T
A
G
T
C
A
G
C
T
A
G
C
T
A
G
T
C
A
G
C
T
A
G
C
T
A
G
T
C
  
Reverse Opposite:  

C
T
A
G
T
C
G
A
C
T
G
A
A
C
T
G
T
C
G
A
C
T
G
A
A
C
T
G
T
C
G
A
C
T
G
A
T
C
A
G
  

|  |  |
| --- | --- |
| p-value: | 1e-296 |
| log p-value: | -6.827e+02 |
| Information Content per bp: | 1.797 |
| Number of Target Sequences with motif | 2281.0 |
| Percentage of Target Sequences with motif | 25.67% |
| Number of Background Sequences with motif | 3787.3 |
| Percentage of Background Sequences with motif | 9.97% |
| Average Position of motif in Targets | 102.8 +/- 53.0bp |
| Average Position of motif in Background | 101.5 +/- 78.6bp |
| Strand Bias (log2 ratio + to - strand density) | -0.0 |
| Multiplicity (# of sites on avg that occur together) | 1.42 |
| Motif File: | file (matrix) reverse opposite |

### Similar de novo motifs found

|  |  |  |  |  |  |  |  |
| --- | --- | --- | --- | --- | --- | --- | --- |
| Rank | Match Score | Redundant Motif | P-value | log P-value | % of Targets | % of Background | Motif file |
| 1 | 0.786 | T A G C G A C T G A C T A T G C G A T C G A C T T G A C C A G T G A C T A G T C G A C T A G C T | 1e-283 | -651.997051 | 15.20% | 3.90% | motif file (matrix) |
| 2 | 0.761 | C G A T A T C G A G C T C G A T G A T C G T A C G A C T A G T C C G A T C A G T G A C T G C A T | 1e-203 | -469.108162 | 11.70% | 3.15% | motif file (matrix) |
| 3 | 0.825 | A G C T A G T C A G C T A G C T A G T C A G C T A G C T A G T C A G C T A G C T A G T C A G C T A G C T A G T C | 1e-184 | -425.845104 | 53.75% | 36.83% | motif file (matrix) |
| 4 | 0.819 | T A C G C G T A A T C G A C T G G T C A C T A G A C T G C T G A T C G A T C A G T C G A | 1e-111 | -257.240545 | 11.25% | 4.52% | motif file (matrix) |
| 5 | 0.777 | C T G A A T G C C G A T A C G T A G T C C G A T T A C G A G T C C G A T G C A T G A C T A G C T | 1e-105 | -242.785873 | 8.22% | 2.78% | motif file (matrix) |
| 6 | 0.775 | C T A G T C G A T C A G C T A G T G C A C T G A A C T G C T G A | 1e-99 | -229.313824 | 18.53% | 10.07% | motif file (matrix) |
| 7 | 0.834 | T A C G T G C A T C A G A C T G G T C A C G T A T A C G T G C A T C G A C T A G A T G C | 1e-77 | -177.421855 | 6.31% | 2.20% | motif file (matrix) |
| 8 | 0.751 | A T G C A C G T A G C T A G T C C G A T A T C G G T A C G C A T C A G T A G T C A G T C C G A T T A G C A G T C | 1e-16 | -38.705525 | 0.96% | 0.26% | motif file (matrix) |
